# Diclofenac Sodium Treatment Ameliorates Extrapancreatic Organ Injuries in a Murine Model of Acute Pancreatitis Induced by Caerulein

**DOI:** 10.1155/2018/9829208

**Published:** 2018-10-31

**Authors:** Ozlem Ozer Cakir, Siddika Findik

**Affiliations:** ^1^Alanya Alaaddin Keykubat University, School of Medicine, Department of Gastroenterology and Hepatology, Turkey; ^2^Necmettin Erbakan University, Meram School of Medicine, Department of Pathology, Turkey

## Abstract

**Aim:**

We determined the effects of diclofenac sodium, octreotide, and their combination on extrapancreatic organ injuries in caerulein-induced acute pancreatitis in mice.

**Methods:**

A total of 58 BALB-C male mice (25 g) were divided into seven groups and used to create a caerulein-induced acute pancreatitis model. Diclofenac sodium, octreotide, and their combination were given for treatment of caerulin-induced acute pancreatitis in mice. At the end of the experiment, the lung, liver, kidney, and stomach were removed for histopathologic assessment.

**Results:**

Histopathologic investigation revealed a statistically significant difference between the groups in mean congestion, edema, tubular injury, perirenal fat tissue inflammation, and tubular stasis scores in kidney tissue (*P* < 0.001, *P* < 0.001, *P* < 0.001, *P* < 0.001, and *P* = 0.048, respectively); mean congestion, edema, neutrophil inflammation, mononuclear inflammation, and emphysematous change scores in the lung (*P* < 0.001, *P* < 0.001, *P* < 0.001, *P* = 0.030, and *P* < 0.001, respectively); mean congestion, edema, and neutrophil inflammation scores in the stomach (*P* = 0.008, *P* = 0.014, and *P* < 0.001, respectively); and mean congestion and hydropic degeneration scores in the liver (*P* = 0.029 and *P* = 0.002, respectively).

**Conclusion:**

Diclofenac sodium alone ameliorates lung edema due to caerulin-induced acute pancreatitis.

## 1. Introduction

Acute pancreatitis is usually diagnosed by the presence of acute abdominal pain and increased serum amylase and lipase levels. Acute pancreatitis may cause damage to other organs because of systemic inflammation and cytokines. In particular, severe acute pancreatitis (SAP) causes other organ damage and has a high mortality rate (range, 15%–40%) [1, 2]. However, mild acute pancreatitis has a lower mortality rate. SAP is seen in approximately 20%–30% of all acute pancreatitis patients [3–5]. The inflammatory cytokines, for example, early inflammatory cytokines (tumor necrosis factor-*α* and interleukin-6), late inflammatory mediator high mobility group box 1 (HMGB1), and reactive oxygen species, have a role in the pathogenesis of SAP [4, 6–8].

Because of these inflammatory cytokines, SAP causes systemic inflammation that results in damage to other organs, such as the lung, liver, kidney, and stomach [3, 6, 9–11]. We used diclofenac sodium, octreotid, and their combination for treatment of caerulein-induced experimental acute pancreatitis in mice and evaluated the effects of these treatments on other organ damage.

## 2. Materials and Methods

We obtained approval from the local ethical committee of the Research and Application Center for Experimental Animals at our university. A total of 58 male BALB-C mice (25 g) were divided into four control and three treatment groups. The control groups were group 1, saline (6 mice); group 2, acute pancreatitis (with intraperitoneal caerulein; 9 mice); group 3, octreotide (6 mice); and group 4, diclofenac sodium (6 mice). The treatment groups were group 5, octreotide with acute pancreatitis (9 mice); group 6, diclofenac sodium with acute pancreatitis (10 mice); and group 7, octreotide plus diclofenac sodium with acute pancreatitis (12 mice). The animals were not used in another study before our model, given standard laboratory foods and drinking water, and housed with a 12 : 12 h light/dark cycle at 21°C–24°C. The animals received only water for 12 h before the beginning of the experiment. All experiments were performed under anesthesia, and the mice were scarified 6 h after the last injection.

### 2.1. Experimental Design

The anesthesia protocol consisted of 2.5 mg xylazine plus 7.5 mg ketamine per 100 g body weight in 200 *μ*l saline (intramuscular). Caerulein (50 *μ*g/kg in 50 *μ*l 0.9% NaCl) was injected intraperitoneally seven times at 1 h intervals under anesthesia to induce acute pancreatitis. Octreotide (250 *μ*g/kg) was injected intraperitoneally 1 h after the first caerulein injection and seven times thereafter at 1 h intervals. Diclofenac sodium (15 mg/kg) was given as a single intraperitoneal injection 1 h after the first caerulein injection. All animals were killed by taking 1–2 ml blood from their hearts 6 h after the last caerulein injection, and their lung, liver, kidney, and stomach were removed immediately.

### 2.2. Histopathologic Examination

#### 2.2.1. Preparing the Tissues

All tissues were fixed in 10% formaldehyde solution for 24 h, processed using the autotechnicon instrument (Leica ASP300; Leica Biosystems, Wetzlar, Germany), and embedded in paraffin blocks. A microtome was used to cut 5 *μ*m sections to obtain slides with lysine, which were stained with hematoxylin–eosin (H&E).

#### 2.2.2. Histopathologic Evaluation

H&E-stained preparations were evaluated by a single experienced pathologist in a blinded fashion using an Olympus BX51 light microscope. Histopathologic criteria were evaluated for the kidney (edema, congestion, intraparenchymal inflammation, perirenal fat tissue inflammation, tubular injury, and tubular stasis), lung (edema, congestion, neutrophil infiltration, mononuclear infiltration, alveolary wall thickness, and emphysematous changes), stomach (edema, congestion, neutrophil infiltration, mononuclear infiltration, and erosion), and liver (congestion, hydropic degeneration, necrosis, portal inflammation, and portal fibrosis). Scores were graded as grades 0 (no pathology), 1 (mild), 2 (moderate), and 3 (severe).

## 3. Statistical Analysis

Data were analyzed using IBM SPSS Statistics version 17.0 software (IBM Corporation, Armonk, NY, USA). Descriptive statistics are shown as the median (25th–75th) percentile for ordinal data. Statistical significance in the differences in histopathologic scores among the groups was evaluated with the Kruskal–Wallis test. When *P* values from the Kruskal–Wallis test were statistically significant (*P* < 0.05), Conover's multiple comparison test was used to determine which group differed from others.

## 4. Results

Histopathologic investigation showed statistically significant differences between the groups in mean congestion, edema, tubular injury, perirenal fat tissue inflammation, and tubular stasis scores in kidney tissue (*P* < 0.001, *P* < 0.001, *P* < 0.001, *P* < 0.001, and *P* = 0.048, respectively; Table 1); congestion, edema, neutrophil inflammation, mononuclear inflammation, and emphysematous change scores in the lung (*P* < 0.001, *P* < 0.001, *P* < 0.001, *P* = 0.030, and *P* < 0.001, respectively; Table 2); congestion, edema, and neutrophil inflammation scores in the stomach (*P* = 0.008, *P* = 0.014, and *P* < 0.001, respectively; Table 3); and congestion and hydropic degeneration scores in the liver (*P* = 0.029 and *P* = 0.002, respectively; Table 4).

### 4.1. Kidney Mean Congestion

Conover's multiple comparison test after the Kruskal–Wallis test in multiple comparisons revealed statistically significant differences in mean congestion scores between group 1 and groups 2 and 5–7 (*P* < 0.001, *P* = 0.003, *P* = 0.007, and *P* = 0.002, respectively). Comparing group 2 with the other groups, the scores were significantly lower in groups 1, 3, and 4 than in group 2 (*P* < 0.001, *P* < 0.001, and *P* = 0,003, respectively). There were no statistically significant differences between groups 1, 3, and 4 (*P* > 0.005). Group 7 (combination group) had higher mean scores than groups 5 and 6, but the difference was not significant (*P* > 0.05).

### 4.2. Kidney Edema

There were significant differences between group 2 and groups 1, 3, and 4 (*P* = 0.004, *P* < 0.001, and *P* < 0.001, respectively) but not between group 2 and groups 5–7 (*P* > 0.05). Comparing group 1 with the other groups showed no significant differences between group 1 and groups 3 and 4 (*P* > 0.05), but there were significant differences between group 1 and groups 5–7 (*P* = 0.002, *P* = 0.017, and *P* = 0.003, respectively).

### 4.3. Kidney Tubular Injury

There were significant differences between group 1 and groups 2 and 5–7 (*P* < 0.001, *P* < 0.001, *P* < 0.001, and *P* < 0.001, respectively) but not between group 1 and groups 3 and 4 (*P* > 0.05). Comparing group 2 and the other groups showed no significant differences between group 2 and groups 3 and 4 (*P* < 0.001 and *P* = 0.002, respectively) or between group 2 and groups 5–7 (*P* > 0.05).

### 4.4. Kidney Perirenal Fat Tissue Inflammation

Significant differences were found between group 1 and groups 2 and 5–7 (*P* < 0.001, *P* < 0.001, *P* < 0.001, and *P* < 0.001, respectively) but not between group 1 and groups 3 and 4 (*P* > 0.05). Comparison of group 2 and the other groups showed a significant difference between group 2 and groups 3 and 4 (*P* < 0.001 and *P* < 0.001, respectively) but not between group 2 and groups 5–7 (*P* > 0.05).

### 4.5. Kidney Tubular Stasis

Comparing group 1 and the other groups showed significant differences between group 1 and groups 2 and 5 (*P* = 0.032 and *P* = 0.043, respectively) but not between group 1 and groups 3, 4, 6, and 7 (*P* > 0.05). Significant differences also were seen between group 2 and groups 3 and 4 (*P* = 0.032 and *P* = 0.032, respectively) but not between group 2 and groups 5–7 (*P* > 0.05). We found lower tubular stasis scores in groups 6 and 7 than in group 5, but this was not statistically significant (*P* > 0.05).

### 4.6. Lung Congestion

Significant differences were found between group 2 and groups 1, 3, 4, 6, and 7 (*P* < 0.001, *P* < 0.001, *P* = 0.019, *P* < 0.001, and *P* = 0.014, respectively) but not between groups 2 and 5 (*P* = 0.153) nor between groups 1 and 3 (*P* = 0.126). However, there were significant differences between group 1 and groups 4–7 (*P* = 0.008, *P* < 0.001, *P* = 0.026, and *P* < 0.001, respectively).

### 4.7. Lung Edema

There were statistically significant differences between group 2 and groups 1, 3, 4, and 6 (*P* = 0.007, *P* = 0.007, *P* = 0.007, and *P* < 0.001, respectively). The lung edema scores in group 6 (diclofenac sodium treatment) were statistically significantly lower than those in groups 5 and 7 (*P* = 0.005 and *P* < 0.001, respectively). A significant difference also was noted between group 1 and groups 2 and 7 (*P* = 0.007 and *P* = 0.010, respectively).

### 4.8. Lung Neutrophil Inflammation

A significant difference was found only between groups 2 and 7 (*P* = 0.044). There were no significant differences in other group comparisons (*P* > 0.05).

### 4.9. Lung Mononuclear Inflammation

There was no significant difference between group 1 and all other groups (*P* > 0.05). A significant difference was found only between groups 2 and 6 (*P* = 0.047). No significant difference was noted between group 2 and the other groups (*P* > 0.05).

### 4.10. Lung Emphysematous Changes

There were statistically significant differences between group 1 and groups 2 and 5–7 (*P* = 0.005, *P* = 0.014, *P* = 0.012, and *P* = 0.010, respectively) but not between group 1 and groups 3 and 4 (*P* > 0.05). Significant differences were noted between groups 2 and 3 (*P* < 0.001).

### 4.11. Stomach Congestion

There were significant differences between group 1 and groups 2 and 5–7 (*P* = 0.003, *P* = 0.046, *P* < 0.001, and *P* = 0.021, respectively). Comparing group 2 and the other groups revealed significant differences between groups 2 and 3 (*P* = 0.014) but not in binary group comparison of the treatment groups (*P* > 0.05).

### 4.12. Stomach Edema

Significant differences were noted between group 1 and groups 2 and 6 (*P* = 0.014 and *P* = 0.005, respectively) but not between group 1 and groups 3–5 and 7 (*P* > 0.05). Comparing group 2 and the other groups showed significant differences between group 2 and groups 3 and 4 (*P* = 0.014 and *P* = 0.054, respectively).

### 4.13. Stomach Neutrophil Inflammation

Statistically significant differences were noted between group 2 and groups 1 and 3–6 (*P* = 0.003, *P* < 0.001, *P* < 0.001, *P* = 0.006, and *P* = 0.006, respectively). When we compared group 7 (combined treatment) and groups 5 and 6, group 7 showed statistically significantly higher scores (*P* = 0.011 and *P* = 0.010, respectively). There was a significant difference between group 1 and groups 2 and 7 (*P* = 0.003 and *P* = 0.005, respectively).

### 4.14. Liver Congestion

Significant differences were noted between groups 1 and 2 (*P* = 0.006) but not between group 1 and the other groups (*P* > 0.05). However, there were significant differences between group 2 and groups 3–7 (*P* < 0.001, *P* = 0.006, *P* = 0.047, *P* = 0.049, and *P* = 0.038, respectively).

### 4.15. Liver Hydropic Degeneration

There were significant differences between group 2 and groups 3–5 and 7 (*P* < 0.001, *P* < 0.001, *P* = 0.040, and *P* = 0.011, respectively) but not between groups 2 and 6 (*P* > 0.05). Significant differences were also found between groups 1 and 2 and 6 (*P* < 0.001 and *P* = 0.048, respectively) but not between group 1 and the other groups (*P* > 0.05).

## 5. Discussion

Acute pancreatitis is a common inflammatory condition of the pancreas and begins as local inflammation. Spread of the inflammation can lead to systemic multiple organ dysfunction and death [12–14]. Multiple organ dysfunction in patients with acute pancreatitis includes many of organ injuries, such as those in the lung, liver, kidney, and stomach. The mortality rate is increased in patients with acute pancreatitis–associated multiple organ dysfunction [12, 14]. The etiopathogenesis of acute pancreatitis–associated extrapancreatic organ injuries and its treatment are not clear. We showed the effects of diclofenac sodium, octreotide, and their combination on extrapancreatic organ injuries (kidney, lung, stomach, and liver) in caerulein-induced acute pancreatitis. To our knowledge, ours is the first study that included these drugs.

A previous study suggested that N-acetylcysteine treatment ameliorated pulmonary and renal tissue damages in all microscopic parameters [15]. Other studies have shown that anti-HMGB1 treatment protected against renal injury in experimental acute pancreatitis [16–18]. In one study, ethyl pyruvate as an anti-inflammatory agent improved extrapancreatic organ injuries in experimental acute pancreatitis [19]. Another study showed that Eugenol administration was protective for the kidneys in an experimental model of acute pancreatitis in rats [20]. One study showed that kynurenine-3-monooxygenase inhibition prevented lung, liver, and kidney injuries in rodent models of acute pancreatitis [21]. Our study showed that congestion, edema, tubular injury, perirenal fat tissue inflammation, and tubular stasis in treatment groups (diclofenac sodium, octerotide, and their combination) were lower than in the acute pancreatitis group, but these differences were not significant.

Heparin administration has been suggested to have a positive influence on lung, liver, kidney, colon, and stomach microcirculatory disturbances accompanying experimental caerulin-induced acute pancreatitis [22]. de Campos et al. [23] showed that pentoxifylline significantly attenuated histologic lung injury, pulmonary neutrophil activity, and proinflammatory signaling in a severe model of acute pancreatitis. A previous study suggested that intravenous selenium given 24 h after induction of experimental acute pancreatitis was associated with a dramatic reduction in bronchoalveolar lavage protein content; therefore, selenium improved lung injury in experimental acute pancreatitis [24]. Another experimental model of acute pancreatitis showed that low-dose dopamine attenuated the lung injury [25]. Multiple antioxidant interventions ameliorated lung injury in experimental acute pancreatitis [26].

In our study, lung congestion scores in groups 6 (diclofenac sodium treatment) and 7 (diclofenac sodium plus octreotide combination) were significantly lower than those in group 2 (acute pancreatitis). Group 6 also showed significantly lower lung edema scores than group 2. Therefore, diclofenac sodium as an anti-inflammatory drug ameliorated lung injury in experimental acute pancreatitis. Neutrophil inflammation of the lung in group 7 was significantly lower than that in group 2 in our study. Also, mononuclear inflammation of the lung was only lower in group 6 than in group 2.

Dobosz et al. [27] concluded that nitric oxide had a beneficial influence on the capillary organ perfusion of the pancreas, liver, colon, stomach, and kidney in experimental acute pancreatitis [27]. Another study showed that nitric oxide also inhibited pancreatitis-induced lung injury [28].

Our study showed that congestion and edema scores for the stomach in all treatment groups were similar to those of group 2. Although neutrophil inflammation of the stomach in group 7 was similar to that in group 2, it was significantly higher than those in the other treatment groups (groups 5 and 6). We concluded that there might be a counteraction between diclofenac sodium and octreotide.

A previous study showed that gadolinium chloride reduced hepatic or systemic cytokine levels and lung injury in experimental pancreatitis [11]. Kiyonari et al. [29] stated that intravenous lidocaine attenuated the lung injury induced by the pancreatic enzymes. Another study showed that 1-week octreotide administration in an experimental acute pancreatitis model was not associated with pathologic changes in digestive organs except the liver [30]. Ohkawara et al. [31] suggested that chlorogenic acid had an anti-inflammatory effect on L-arginine-induced pancreatitis and pancreatitis-associated lung injury. Recombinant human hepatocyte growth factor provided protective effects for lung injury in caerulein-induced acute pancreatitis in mice [32].

In our study, liver congestion in all treatment groups was significantly lower than that in the acute pancreatitis group. In terms of hydropic degeneration of the liver, the diclofenac sodium treatment group was similar to the acute pancreatitis group and had significantly higher levels than the other treatment groups. However, the octreotide and combination treatment groups showed significantly lower levels than the acute pancreatitis group in terms of hydropic degeneration of the liver. The prognosis of acute pancreatitis is associated with extrapancreatic organ injuries. Also, the treatment of this situation is not clear. We showed the effects of diclofenac sodium, octreotide, and their combination on extrapancreatic organ injuries (kidney, lung, stomach, and liver) in caerulein-induced acute pancreatitis in mice. Our study demonstrated that diclofenac sodium improved experimental acute pancreatitis–associated lung edema. Diclofenac sodium may ameliorate the prognosis of acute pancreatitis by reducing lung edema.

## 6. Conclusions

In conclusion, diclofenac sodium is a well-known drug that may be a good choice for extrapancreatic organ injuries in cases of acute pancreatitis. Diclofenac sodium improved lung edema–associated experimental acute pancreatitis in our study. Therefore, diclofenac sodium may improve the prognosis of acute pancreatitis by reducing lung edema. Further studies are needed for clarity.

## Figures and Tables

**Table 1:** Histopathological results obtained from the kidney tissue according to groups.

	Group 1 (*n* = 6)	Group 2 (*n* = 9)	Group 3 (*n* = 6)	Group 4 (*n* = 6)	Group 5 (*n* = 9)	Group 6 (*n* = 10)	Group 7 (*n* = 12)	*P* value^†^
Congestion	0 (0–1)	2 (1–2.5)^a^	0 (0–1)^b^	0.5 (0–1)^b^	1 (1–2)^a,c,d^	1 (1–2)^a,c,d^	1.5 (1–2)^a,c,d^	**<0.001**
Edema	0 (0–1)	1 (1–2.5)^a^	0 (0–0.25)^b^	0 (0–0)^b^	1 (1–2)^a,c,d^	1 (1–2)^a,c,d^	1 (1–2)^a,c,d^	**<0.001**
Tubular injury	0 (0–0)	1 (1–2)^a^	0 (0–0.25)^b^	0 (0–1)^b^	1 (1–2)^a,c,d^	1 (1–2)^a,c,d^	1 (1–2)^a,c,d^	**<0.001**
Parenchymal inflammation	0 (0–0)	0 (0–0)	0 (0–0)	0 (0–0)	0 (0–0)	0 (0–0)	0 (0–0)	0.253
Perirenal fat tissue inflammation	0 (0–0)	2 (1.5–3)^a^	0 (0–0)^b^	0 (0–0)^b^	2 (1–2)^a,c,d^	1.5 (1–2)^a,c,d^	1 (1–2)^a,c,d^	**<0.001**
Tubular stasis	0 (0–0)	1 (0–1.5)^a^	0 (0–0)^b^	0 (0–0)^b^	1 (0–1)^a,c,d^	0 (0–1)	0 (0–1)	**0.048**

Data were shown as median (25th–75th) percentiles, the Kruskal–Wallis test. ^a^The difference between the considered group and group 1 was found as statistically significant (*P* < 0.05). ^b^The difference between the considered group and group 2 was found as statistically significant (*P* < 0.05). ^c^The difference between the considered group and group 3 was found as statistically significant (*P* < 0.05). ^d^The difference between the considered group and group 4 was found as statistically significant (*P* < 0.05).

**Table 2:** Histopathological results obtained from the lung tissue according to groups.

	Group 1 (*n* = 6)	Group 2 (*n* = 9)	Group 3 (*n* = 6)	Group 4 (*n* = 6)	Group 5 (*n* = 9)	Group 6 (*n* = 10)	Group 7 (*n* = 12)	*P* value^†^
Congestion	0 (0–0.25)	2 (2–2)^a^	1 (0–1.25)^b^	1 (1–2)^a,b^	2 (1–2)^a,c^	1 (1–1)^a,b,d^	1 (1–2)^a,b^	**<0.001**
Edema	0 (0–1)	1 (1–1)^a^	0 (0–1)^b^	0 (0–1)^b^	1 (0–1)	0 (0–0)^b,d^	1 (1–1)^a,c,e,f^	**<0.001**
Neutrophil infiltration	0 (0–0)	0 (0–1)	0 (0–0)	0 (0–0)	0 (0–0)	0 (0–0)	0 (0–0)^b^	**<0.001**
Mononuclear infiltration	0 (0–0)	0 (0–1.5)	0 (0–0)	0 (0–0)	0 (0–0.5)	0 (0–0)^b^	0 (0–0)	**0.030**
Alveolary wall thickness	0 (0–0)	0 (0–0)	0 (0–0)	0 (0–0)	0 (0–0)	0 (0–0)	0 (0–0)	>0.999
Emphysematous changes	0 (0–1)	1 (1–1)^a^	0 (0–0)^b^	1 (0.75–1)^c^	1 (1–1)^a,c^	1 (1–1)^a,c^	1 (1–1)^a,c^	**<0.001**

Data were shown as median (25th–75th) percentiles, the Kruskal–Wallis test. ^a^The difference between the considered group and group 1 was found as statistically significant (*P* < 0.05). ^b^The difference between the considered group and group 2 was found as statistically significant (*P* < 0.05). ^c^The difference between the considered group and group 3 was found as statistically significant (*P* < 0.05). ^d^The difference between the considered group and group 5 was found as statistically significant (*P* < 0.05). ^e^The difference between the considered group and group 4 was found as statistically significant (*P* = 0.010). ^f^The difference between the considered group and group 6 was found as statistically significant (*P* < 0.001).

**Table 3:** Histopathological results obtained from the stomach tissue according to groups.

	Group 1 (*n* = 6)	Group 2 (*n* = 9)	Group 3 (*n* = 6)	Group 4 (*n* = 6)	Group 5 (*n* = 9)	Group 6 (*n* = 10)	Group 7 (*n* = 12)	*P* value^†^
Congestion	0 (0–0.25)	1 (1–1.5)^a^	0 (0–1)^b^	1 (0–1)	1 (0–1)^a^	1 (1–1.25)^a,c^	1 (0.25–1)^a^	**0.008**
Edema	0 (0–1)	1 (1–1.5)^a^	0 (0–1)^b^	0.5 (0–1)	1 (0.5–1)	1 (1–1.25)^a,c,d^	1 (0.25–1)	**0.014**
Neutropil infiltration	0 (0–0.25)	1 (1–2)^a^	0 (0–0)^b^	0 (0–0)^b^	0 (0–1)^b^	0 (0–1)^b^	1 (1–1)^a,c,d,e,f^	**<0.001**
Erosion	0 (0–0)	0 (0–0)	0 (0–0)	0 (0–0)	0 (0–0)	0 (0–0)	0 (0–0)	>0.999
Mononuclear infiltration	0.5 (0–1)	1 (0.5–1)	0.5 (0–1)	1 (0–1)	1 (0–1.5)	1 (1–1)	1 (1–1)	0.272

Data were shown as median (25th–75th) percentiles, the Kruskal–Wallis test. ^a^The difference between the considered group and group 1 was found as statistically significant (*P* < 0.05). ^b^The difference between the considered group and group 2 was found as statistically significant (*P* < 0.05). ^c^The difference between the considered group and group 3 was found as statistically significant (*P* < 0.01). ^d^The difference between the considered group and group 4 was found as statistically significant (*P* < 0.05). ^e^The difference between the considered group and group 5 was found as statistically significant (*P* = 0.011). ^f^The difference between the considered group and group 6 was found as statistically significant (*P* = 0.010).

**Table 4:** Histopathological results obtained from the liver tissue according to groups.

	Group 1 (*n* = 6)	Group 2 (*n* = 9)	Group 3 (*n* = 6)	Group 4 (*n* = 6)	Group 5 (*n* = 9)	Group 6 (*n* = 10)	Group 7 (*n* = 12)	*P* value^†^
Congestion	0 (0–1)	1 (1–1.5)^a^	0 (0–0.25)^b^	0 (0–1)^b^	1 (0–1)^b^	1 (0–1)^b^	1 (0–1)^b^	**0.029**
Hydropic degeneration	0 (0–1)	2 (1–2)^a^	0 (0–0.25)^b^	0 (0–1)^b^	1 (0–1.5)^b,c^	1 (0.75–1.25)^a,c,d^	1 (0–1)^b^	**0.002**
Focal necrosis	0 (0–0)	0 (0–1)	0 (0–0.25)	0 (0–0)	0 (0–0.5)	0 (0–0.25)	0 (0–0)	0.640
Portal inflammation	0 (0–0)	0 (0–1)	0 (0–0)	0 (0–0)	0 (0–0.5)	0 (0–1)	0 (0–0)	0.259
Portal fibrosis	0 (0–0)	0 (0–0)	0 (0–0)	0 (0–0)	0 (0–0)	0 (0–0)	0 (0–0)	>0.999

Data were shown as median (25th–75th) percentiles, the Kruskal–Wallis test. ^a^The difference between the considered group and group 1 was found as statistically significant (*P* < 0.05). ^b^The difference between the considered group and group 2 was found as statistically significant (*P* < 0.05). ^c^The difference between the considered group and group 3 was found as statistically significant (*P* < 0.05). ^d^The difference between the considered group and group 4 was found as statistically significant (*P* = 0.048).

## Data Availability

The data used to support the findings of this study are available from the corresponding author upon request.

## References

[1] Carroll J. K., Herrick B., Gipson T., Lee S. P. (2007). Acute pancreatitis: diagnosis, prognosis, and treatment. *American Family Physician*.

[2] Norkina O., Graf R., Appenzeller P., De Lisle R. C. (2006). Caerulein-induced acute pancreatitis in mice that constitutively overexpress Reg/PAP genes. *BMC Gastroenterology*.

[3] Yang Z., Meng X., Xu P. (2015). Central role of neutrophil in the pathogenesis of severe acute pancreatitis. *Journal of Cellular and Molecular Medicine*.

[4] Luan Z. G., Ma X. C., Zhang H., Zhang C., Guo R. X. (2013). Protective effect of ethyl pyruvate on pancreas injury in rats with severe acute pancreatitis. *The Journal of Surgical Research*.

[5] Hegazi R., Raina A., Graham T. (2011). Early jejunal feeding initiation and clinical outcomes in patients with severe acute pancreatitis. *Journal of Parenteral and Enteral Nutrition*.

[6] Yang R., Uchiyama T., Alber S. M. (2004). Ethyl pyruvate ameliorates distant organ injury in a murine model of acute necrotizing pancreatitis. *Critical Care Medicine*.

[7] Yang Z.-Y., Ling Y., Yin T. (2008). Delayed ethyl pyruvate therapy attenuates experimental severe acute pancreatitis via reduced serum high mobility group box 1 levels in rats. *World Journal of Gastroenterology*.

[8] Cheng B.-Q., Liu C. T., Li W. J. (2007). Ethyl pyruvate improves survival and ameliorates distant organ injury in rats with severe acute pancreatitis. *Pancreas*.

[9] Akbarshahi H., Rosendahl A. H., Westergren-Thorsson G., Andersson R. (2012). Acute lung injury in acute pancreatitis-awaiting the big leap. *Respiratory Medicine*.

[10] Yang R., Zou X., Tenhunen J. (2014). HMGB1 neutralization is associated with bacterial translocation during acetaminophen hepatotoxicity. *BMC Gastroenterology*.

[11] Gloor B., Blinman T. A., Rigberg D. A. (2000). Kupffer cell blockade reduces hepatic and systemic cytokine levels and lung injury in hemorrhagic pancreatitis in rats. *Pancreas*.

[12] Vasilescu C., Taşcă C. (1991). Acute experimental pancreatitis--morphological evidence for the development of a multiple organ failure syndrome. *Romanian Journal of Morphology and Embryology*.

[13] Lowenfels A. B., Maisonneuve P., Sullivan T. (2009). The changing character of acute pancreatitis: epidemiology, etiology, and prognosis. *Current Gastroenterology Reports*.

[14] Pastor C. M., Matthay M. A., Frossard J. L. (2003). Pancreatitis-associated acute lung injury: new insights. *Chest*.

[15] Atayoğlu K., Gürleyik G., Demirel G., Özkara S. (2017). Effect of N-acetylcysteine on neutrophil functions during experimental acute pancreatitis. *Ulusal Travma ve Acil Cerrahi Dergisi*.

[16] Lau A., Wang S., Liu W., Haig A., Zhang Z. X., Jevnikar A. M. (2014). Glycyrrhizic acid ameliorates HMGB1-mediated cell death and inflammation after renal ischemia reperfusion injury. *American Journal of Nephrology*.

[17] Hu Y.-M., Pai M. H., Yeh C. L., Hou Y. C., Yeh S. L. (2012). Glutamine administration ameliorates sepsis-induced kidney injury by downregulating the high-mobility group box protein-1-mediated pathway in mice. *American Journal of Physiology-Renal Physiology*.

[18] Sawa H., Ueda T., Takeyama Y. (2006). Blockade of high mobility group box-1 protein attenuates experimental severe acute pancreatitis. *World Journal of Gastroenterology*.

[19] Yang R., Zhu S., Tonnessen T. I. (2016). Ethyl pyruvate is a novel anti-inflammatory agent to treat multiple inflammatory organ injuries. *Journal of Inflammation*.

[20] Markakis C., Tsaroucha A., Papalois A. E. (2016). The role of eugenol in the prevention of acute pancreatitis-induced acute kidney injury: experimental study. *HPB Surgery*.

[21] Mole D. J., Webster S. P., Uings I. (2016). Kynurenine–3–monooxygenase inhibition prevents multiple organ failure in rodent models of acute pancreatitis. *Nature Medicine*.

[22] Dobosz M., Mionskowska L., Hac S., Dobrowolski S., Dymecki D., Wajda Z. (2004). Heparin improves organ microcirculatory disturbances in caerulein-induced acute pancreatitis in rats. *World Journal of Gastroenterology*.

[23] de Campos T., Deree J., Martins J. O. (2008). Pentoxifylline attenuates pulmonary inflammation and neutrophil activation in experimental acute pancreatitis. *Pancreas*.

[24] Hardman J., Jamdar S., Shields C., McMahon R., Redmond H. P., Siriwardena A. K. (2005). Intravenous selenium modulates L-arginine-induced experimental acute pancreatitis. *Journal of the Pancreas*.

[25] Kaya E., Arslan A., Aliyazicioglu Y., Güven H., Aydin O., Ozkan K. (2005). Low dose dopamine prevents end organ damage in experimentally induced pancreatitis. *Hepato-Gastroenterology*.

[26] Hardman J., Shields C., Schofield D., McMahon R., Redmond H. P., Siriwardena A. K. (2005). Intravenous antioxidant modulation of end-organ damage in L-arginine-induced experimental acute pancreatitis. *Pancreatology*.

[27] Dobosz M., Hać S., Wajda Z. (1996). Does nitric oxide protect from microcirculatory disturbances in experimental acute pancreatitis in rats?. *International Journal of Microcirculation, Clinical and Experimental*.

[28] O'Donovan D. A., Kelly C. J., Abdih H. (1995). Role of nitric oxide in lung injury associated with experimental acute pancreatitis. *The British Journal of Surgery*.

[29] Kiyonari Y., Nishina K., Mikawa K., Maekawa N., Obara H. (2000). Lidocaine attenuates acute lung injury induced by a combination of phospholipase A2 and trypsin. *Critical Care Medicine*.

[30] Soran A., Cete M., Cöl C. (1998). The effect of short-term octreotide administration on the histologic structure of stomach, duodenum, jejunum, colon, liver and gallbladder in an experimental pancreatitis model. *The Tohoku Journal of Experimental Medicine*.

[31] Ohkawara T., Takeda H., Nishihira J. (2017). Protective effect of chlorogenic acid on the inflammatory damage of pancreas and lung in mice with l-arginine-induced pancreatitis. *Life Sciences*.

[32] Palestino-Domínguez M., Pelaez-Luna M., Lazzarini-Lechuga R. (2018). Recombinant human hepatocyte growth factor provides protective effects in cerulein-induced acute pancreatitis in mice. *Journal of Cellular Physiology*.

